# How to Implement Robots in Interventions for Children with Autism? A Co-creation Study Involving People with Autism, Parents and Professionals

**DOI:** 10.1007/s10803-017-3235-9

**Published:** 2017-07-06

**Authors:** Claire A. G. J. Huijnen, Monique A. S. Lexis, Rianne Jansens, Luc P. de Witte

**Affiliations:** 1 Research Centre Technology in Care, Zuyd University of Applied Sciences, Henri Dunantstraat 2, 6419 PB Heerlen, The Netherlands; 2 CAPHRI School for Public Health and Primary Care, Faculty of Health, Medicine and Life Sciences, Maastricht University, Maastricht, The Netherlands; 3 Occupational Therapy Department, Zuyd University of Applied Sciences, Heerlen, The Netherlands; 4 CATCH Centre for Assistive Technology and Connected Healthcare, University of Sheffield, Sheffield, UK

**Keywords:** Autism, Robot, Implementation, Robot mediated intervention, KASPAR, Co-creation, Requirements, Robot assisted intervention

## Abstract

The aim of this study was to gain insight into how robots can be practically implemented into current education and therapy interventions for children with autism spectrum disorder (ASD). This qualitative study included focus groups and co-creation sessions. 73 Participants (professionals and adults with ASD) took part in 13 focus groups to elicit requirements for robot assisted interventions. Additionally, 22 participants (professionals, parents of children with ASD and adults with ASD) generated ideas for interventions using robot KASPAR in three co-creation sessions. This study resulted in: an overview of requirements concerning the robot, end-user, environment and practical implementation; a template to systematically describe robot interventions in general and for KASPAR in particular; and finally new interventions.

## Introduction

Personalised and early therapy and intervention plans are effective in supporting individuals to cope with autism spectrum disorder (ASD) associated symptoms (Volkmar et al. 2005). The call for early empowerment of people with ASD relates to the vision of the World Health Organization (WHO), who recently redefined its meaning of disability as the result of the person’s interaction with his environment. They argue that it is “an evolving concept”, and “disability results from the interaction between persons with impairments and attitudinal and environmental barriers that hinder their full and effective participation in society on an equal basis with others” (WHO 2011). They also state that social participation can be improved when the barriers are addressed that hinder people with disabilities in their daily lives. Assistive technologies, when designed and implemented appropriately, and meeting the needs of the user and their environment, are powerful tools to boost independence and improve participation (WHO 2011). A variety of assistive applications are suggested for people with ASD, to support them in varies areas of their life, and are implemented in computers, special input devices, virtual environments, avatars, serious games, tele rehabilitation as well as robots (Boucenna et al. 2014). Moreover, an increasing sophistication and transformation can be seen from ASD technology research mainly as theoretical novelties, now growing towards “tools that are better understood, more solidly studied, more nuanced, and more practically relevant” (Shic and Goodwin 2015). Various publications and studies highlight the potential and state of the art of using robots as assistive tools in interventions for children with ASD (Diehl et al. 2012; Huijnen et al. 2016a).

Interacting with robots can be particularly empowering for children with ASD, because it may overcome various barriers experienced in face-to-face interaction with humans. Moreover, robot assisted interventions can be tailored to the needs of the specific child and can be used in an identical manner as often as needed. However, the actual implementation and daily use of robots in practice is still not very common. Begum et al. (2016) suggest a roadmap to establish robot-mediated interventions as an evidence based practice (EBP) in the domain of autism, since EBP has become a benchmark in ASD intervention. In order to increase the applicability for clinical practitioners, they propose a number of guidelines, based on a comprehensive review of clinical literature on ASD interventions, for human-robot interaction (HRI) studies on robot-mediated interventions (RMI). These elements are: a clear description of the goal of the intervention, the participants, independent variables with RMI, dependent variables, research design as well as generalization training (Begum et al. 2016).

**Fig. 1:**
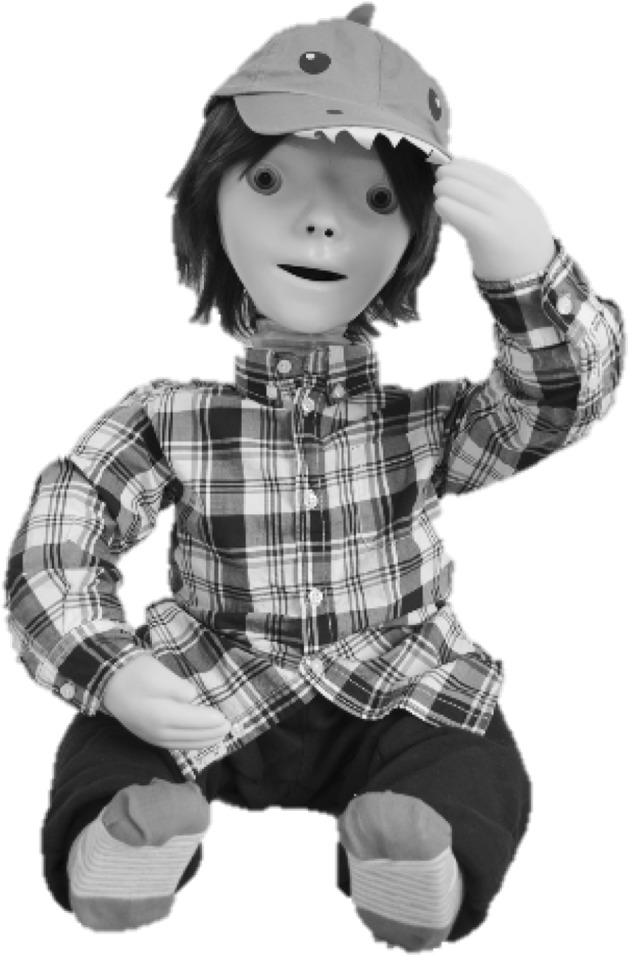
Therapy robot KASPAR

One of the robot platforms used in various (HRI) studies in education/therapy settings is KASPAR (“Kinesics and Synchronization in Personal Assistant Robotics”) (Dautenhahn et al. 2003, 2009; Huijnen et al. 2016a; Robins et al. 2010; Wainer et al. 2010, 2014a). KASPAR is a semi-autonomous minimally invasive humanoid robot developed by the Adaptive Systems Group of the University of Hertfordshire (UK) (see Fig. 1).

KASPAR allows for several operation modes:

1. automatic behaviour or autonomous control when its sensors (e.g. on KASPAR’s head, torso, arms, hands, feet) are activated by the child, as well as
2. as a controlled operating mode used by the accompanying professional or a child by means of a remote control for KASPAR, and/or
3. a combination of both which creates a semi-autonomous mode of KASPAR.

Customisation software allows for the creation of new (personalised) KASPAR scenarios. This customisation software enables users to make new KASPAR poses, behaviour, speech or sounds and scenarios and to fine-tune or personalise existing ones. It can be installed on any regular laptop on which the KASPAR application is running.

Studies indicate that KASPAR can contribute to positive results in the area of increasing body awareness, encouraging collaborative skills (Wainer et al. 2014b); prolonging children’s attention span (Costa et al. 2013); mediating and encouraging social interaction (Ben Robins et al. 2009); and learning appropriate physical interaction (Costa et al. 2015).

Professionals see a clear potential for KASPAR for a relative high number of education and/or therapy goals for this target group (Huijnen et al. 2016a, b). This applies not only to the more commonly addressed challenging areas such as social interaction and communication, but also in objectives related to preschool skills, play and emotional wellbeing for example (Huijnen et al. 2016a, b). Examples are: ‘pose a question’, ‘ask for help’, ‘imitation in play’, ‘making contact’, ‘follow up instructions’ and ‘having fun’.

In order to go beyond “likeability” of robots for children with autism as Begum et al. (2016) state it, we decided to intensely involve clinicians and practitioners in the process of actually creating new robot mediated interventions themselves. This facilitates the step from moving from isolated lab HRI studies towards feasibility and effect studies, integrated EBP and application of the use of robots in autism interventions that children actually benefit from in their life. This entails not only creating (the contents of) the robot, in this case KASPAR, but especially to better understand hów to embed the robot in interventions in practices of the envisioned end-users.

The aim of the current study was to examine how robot KASPAR can be included in interventions to contribute to reaching therapy and educational goals of professionals for children with ASD as well as to create a template that can be used to create and describe new robot interventions, including the human-robot interaction. This article entails to address the following research questions:

1. What are important requirements regarding the implementation of robots such as KASPAR as tools in an ASD intervention?
2. What are important elements in a robot-mediated intervention description and how should the intervention template look like to enable professionals to create new interventions?

## Method

The qualitative study started with focus group sessions to identify intervention requirements as well as crucial elements of an intervention template, followed by co-creation sessions that applied these insights in order to enable professionals and stakeholders to create new robot interventions for robot KASPAR. The importance of understanding people’s thoughts, drivers, challenges and ideas was the reason for choosing these qualitative methods.

### Focus Groups

#### Procedure

The focus groups addressed two main topics:

1. identifying the potential of robot KASPAR and the roles it can take in interventions for children with ASD; and
2. eliciting requirements for robot mediated interventions.

The current paper presents the results of the second topic. The findings regarding the potential and roles of KASPAR are published elsewhere (Huijnen et al. 2017). Part of those results—possible roles for KASPAR such as for example “provoker”, “reinforcer” or “trainer”—are used in the intervention template that is used in the co-creation sessions which will be discussed later in more detail.

In total, 13 focus group sessions of 2 hours each were organised. Two researchers guided the session; one in the role of moderator and one as a note taker, observer and transcriber of the sessions. Researchers collected informed consent forms for making audio recordings, pictures as well as participant information sheets for gathering demographic information. After a welcome, KASPAR was demonstrated. During this demo, KASPAR greeted the participants, played a song and KASPAR’s possibilities were illustrated in an interactive fashion (e.g. the use of sensors, motors, speech, sounds, the remote control and the option to create new personalised scenarios). After the demo, the discussion on requirements started by asking a general question: what are the requirements of a meaningful KASPAR intervention? People were asked to think about aspects required for a successful implementation of a KASPAR intervention. If participants got stuck in the discussion researchers used prompting.. The specific prompts used in the focus groups were: “child”, “professional”, “environment/room/setting”, “KASPAR”, and “school as an entity”.

#### Setting and Participants

Of the 13 sessions; 12 sessions consisted of a group of professionals and 1 session included individuals with ASD. The professionals work with children with ASD at special needs schools, (youth and child) care organisations, pedagogic organisations, ASD treatment centres and medical day care centres in The Netherlands.

The sessions were conducted at the venues of the organisations and the session with participants with autism took place at a meeting room of the Zuyd University of Applied Sciences. Organising these sessions at the venue of the organisation themselves created a familiar and trusted atmosphere for the participants to facilitate free and open minded discussions. The research team prepared the room in advance, creating a U-shape setup to facilitate interaction between participants and preparing beamer/projection facilities in the front for the demonstration of KASPAR.

The background of the professionals was multidisciplinary: ASD teachers, assistants at special need schools, speech therapists, psychologists, physiotherapist, occupational therapists, psychomotor therapist, treatment coordinator, case managers, behavioural therapists and people working in care management functions. The average work experience was 14 years with a standard deviation of 9.5 years. Table 1 presents the characteristics of the 73 participants of the focus group sessions.

**Table 1:** Demographic characteristics of the participants of the focus groups

Description	Value	
	n	(%)
Gender		
Male	13	(18)
Female	60	(82)
Background		
Professional working with children with ASD	70	(97)
Parent of child with ASD	3	(4)
Adult with ASD	3	(4)
Number of years working experience with ASD (years)		
0–5	15	(21)
6–10	19	(26)
11–15	10	(14)
16–20	14	(19)
21–25	3	(4)
26–30	5	(6)
31–35	4	(5)

#### Data Collection

The completed participants demographic forms were collected on paper and the data was imported in an Excel sheet. Audio recordings were made during the sessions (after obtaining informed consent of the participants). One of the researchers present at the sessions literally transcribed all the data of the 13 sessions in Word files. Subsequently, the software program Nvivo was used to transform the data into written text to be able to start the analysis.

#### Data Analysis

For the analysis of the qualitative data of the focus groups, researchers applied conventional content analysis (Hsieh and Shannon 2005). This means that coding categories are directly derived from the data of the focus groups in an inductive manner, rather than from an existing predefined coding scheme. To ensure data integrity and validity we used data triangulation. More than one person was involved in the collection and analysis of the data and multiple methods of data collection were used. The two researchers who also accompanied the sessions created the coding scheme based on analysis of the transcripts. Both researchers read two of the sessions in order to identify main labels to come up with a tentative coding scheme. Thus, a code was assigned to a text chunk of any size that represented a single requirement that was mentioned. The collection of these codes resulted in a coding scheme. Subsequently, an analytical session between the two researchers was organised to compare, discuss, fine-tune and align these two coding schemes to make sure the codes were clear, mutually exclusive and that both researchers had the same understanding of each code. In case of difference, discussion was used to reach consensus. The main researcher then used the resulting intermediate coding scheme was to code sessions 3–5. Subsequently, the two researchers participated in an additional analytical session to check the work again and the final coding scheme was established and applied to the entire corpus of text by the main researcher.

When all the text was analysed and the requirements were obtained, the two researchers constructed the intervention template based on these requirements and insights of the focus group sessions. This template entails main elements to describe in a robot assisted intervention. The template consists of elements such as: “name of the intervention”, “focus on objectives (select from a given set or choose another one)”, “role(s) of KASPAR”, “goal of the session”, “session characteristics” as well as a schematic representation for the envisioned interaction between the professional, KASPAR and the child. For all the elements and the format of the intervention template we refer to “Intervention Template” and a detailed example in “Appendix 2”.

### Co-creation Sessions

The aim of the co-creation sessions was to create new robot interventions, incorporating the identified requirements of the focus groups, in a multidisciplinary group of participants.

#### Procedure

Three co-creation sessions had a duration of 2–3 hours each. After a short welcome, an introduction round to briefly introduce oneself to the others (name, background and current relation to ASD), an explanation of the aim and nature of the session, and a presentation of the intervention template, an interactive live demonstration of KASPAR was given. Participants provided informed consent for making audio recordings during the sessions. After answering questions, participants were asked to think about a certain child with ASD that they have experience with, and to create a meaningful KASPAR intervention using the template. Participants then split up in smaller subgroups to work on a new intervention. The allocation of people to groups was done in a rather organic manner; people seemed to gather around a common theme/idea rather easily; so the creation of groups happened spontaneously based on the topic for the intervention. In all the subgroups, a researcher was present to guide the brainstorm process and answer potential questions. At the end of the session the subgroups gathered around the big table again and presented their interventions to the entire group in order to receive feedback and suggestions for improvement of everybody. Every group delivered at least 1 new intervention for robot KASPAR based on and written down on the intervention template.

#### Setting and Participants

The three sessions where organised at an inspiring venue. The session started with a welcome and introduction part in a group setting. After the introduction, people started the group work on different dedicated tables in the same room. The composition of the groups (session 1 n = 9; session 2 n = 5; session 3 n = 8 participants) was multidisciplinary; such as professionals being teachers or ASD therapists/caregivers, individuals with ASD, parents of children with ASD and partners of people with ASD. In total 22 people participated (see Table 2 for participant characteristics).

**Table 2:** Demographic characteristics of the participants of the co-creation session

Description/variables	Value	
	n	(%)
Gender		
Male	8	(36)
Female	14	(64)
Background		
Professional working with children with ASD	15	(68)
Parent of child with ASD	2	(9)
Partner of person with ASD	1	(4.5)
Adult with ASD	4	(18)
Number of years working/experience with ASD		
0–3 years	2	(9)
3–5 years	1	(4.5)
5–10 years	6	(27)
>10 years	13	(59)

#### Data Collection

During the co-creation sessions, the participants filled in the intervention templates. Researchers collected and digitalised these templates (ten in total) after the session to prepare them for the analysis and further implementation as actual scenarios to be developed in the actual robot.

#### Data Analysis

The person who accompanied that particular subgroup discussion during the session performed the data analysis, not to miss the context and depth of the discussions that took place when creating these interventions. The data analysis was rather straightforward for these interventions since no actual analysis took place on the content of the interventions, but merely an understanding was necessary in order to implement these into the robot platform.

## Results

First the requirements for KASPAR mediated interventions and the intervention template are presented and subsequently the new KASPAR interventions made during the co-creation sessions are described.

### Requirements for KASPAR Mediated Interventions

The intention was to elicit factors that are crucial for a meaningful intervention (practical implementation) rather than ‘only’ understanding technical robot requirements. As a result, the focus groups delivered a number of different categories of requirements for KASPAR mediated interventions. Table 3 summarises the overall categories. The following sections present each of these separate categories in more detail.

**Table 3:** Overview of requirement categories for robot assisted interventions

Requirements overview robot assisted interventions
The robot (KASPAR)
Appearance
Voice and sound
Operation of KASPAR
Behaviour and actions of KASPAR
KASPAR’s attributed / toolbox
The target group
Specification of the target group who will probably benefit from KASPAR
Specification of the target group who will probably not benefit from KASPAR
Environment
Professional
Intervention implementation and integration into common practices
Integration in individualised education/therapy plan
Integration in organisation, professional levels and connecting to parents
Phase in the intervention trajectory
Session characteristics

#### The Robot KASPAR

This section presents the requirements with respect to various aspects of the robot.

##### Appearance

KASPAR’s looks were important according to professionals. They mentioned that KASPAR needs to look cool. He needs to get a set of cool and nice different clothes suitable for different seasons and weather types. When choosing clothes they mentioned to pay attention to the texture of and prints on the clothes. Some children might get an overload if there are a lot of colours, prints or images on clothes. Moreover, KASPAR needs attributes as well that can boost a cool image that children can identify with, such as a cap (that he could wear backwards) and maybe (sun) glasses. When the lesson in the classroom starts, KASPAR has to take of his cap and sunglasses, just like the children. By changing KASPAR’s clothes one can use him in a different context or different role. Some professionals asked if they can change his hairdo and some asked if there is also a girl version of KASPAR.

With this hairdo he could be a girl, while he has the voice of a boy.—Adult with autism, who has a partner and children with autism.

##### Voice and Sound

A few professionals indicated that for some children it would be good if KASPAR would get the voice of a recognisable person for them (e.g. mum or dad) in the beginning and that KASPAR would then get another voice later. However, more professionals suggested to use an artificial voice from the start. This can be a computerised voice as long if this does not sound too artificial, jerky, canny or robot-like. This voice should be soft in nature and sound friendly not to scare them. The sentences KASPAR utters should be short to increase understanding. The talking speed should be rather slow. If the children make a mistake, or if something does not go as well as hoped, KASPAR should give a positive reaction in a neutral voice, without an angry tone (i.e. *please try this again*). If KASPAR will use other sounds than his own voice (e.g. a song), he should always announce this first using his own voice (i.e. *I will now play a song for you)*.

Many children with autism are sensitive for audio or sound. KASPAR could be used to familiarise them with unusual sounds so that they are better prepared or less scared when they hear the sound in the ‘real’ situation (i.e. when they go on a trip outside, first time holiday, visit an animal farm, heavy traffic, trains, yelling children, sirens, clock ticking, or sounds with a high pitch). Many professionals mentioned their expectations with respect to the use of all kinds of songs. This will give them joy and might very well stimulate interaction between children, since many children love music and react positively to it.

Parents imitate unfamiliar sounds as well. What sound does the dog make? Waf, Waf. And the cat? Miauw, miauww…I think KASPAR should indicate that he will produce these sounds.—Adult with autism, who has a partner and children with autism

Imagine a child goes on a school trip. KASPAR can say “I heard you are going on a school trip, I know some sounds that you will hear there, shall I let you hear them?—Adult with autism

##### Operation of KASPAR

Professionals indicated that they need some training to be able to operate KASPAR during the session and to be able to create new scenario’s tailored to the specific needs of a child. They indicated that it should not take a lot of time to get KASPAR up and running before a session since they are already very busy with many things in a classroom or therapy setting. Also they expressed relevance for being flexible in changing KASPAR’s settings or behaviour when the situation demands improvisation. The behaviour of children can hardly be predicted. It is desirable to be able to fine-tune some KASPAR aspects rather easy and quickly if needed.

##### Behaviour and Actions of KASPAR

Professionals indicated that KASPAR should behave in a consistent, clear, playful and accessible manner of interacting with children. When KASPAR moves his body parts, one can hear a sound of the motors being activated. Professionals expected that this might distract some children. They suggested not to use too much movement and speech at the same time since this might cause an information overload for children. KASPAR should either move or speak first, then do the other thing, so that children only have to pay attention to one aspect at the same time. In this sense, the amount of simultaneous stimuli needs to be limited as much as possible.

Some professionals suggested it would be good if KASPAR could fetch, grasp, manipulate or hold objects. In that case he would be able to make a difference between pinching and caressing a cheek for example. At this moment KASPAR’s fingers are fixed, they cannot move separately from each other. It was suggested a number of times that it would be interesting if KASPAR could make gestures and use sign language rather than verbal commands only since quite a share of these children have no or limited verbal skills. KASPAR could then also point to things. Similar as with the use of sounds, KASPAR should not start to move in a very abrupt fast manner as it might scare children. If he was sitting without moving for a while, the start of the movement should be gradual and smooth.

##### KASPAR’s Attributes/Toolbox

Professionals had ideas to integrate KASPAR in other activities or give him objects or tools that they can work with. They mentioned KASPAR could get different attributes such as glasses, or musical instruments and use materials from other methods. For example, by dressing KASPAR up in a police uniform, he gets a different role as when he wears a sporty outfit.

#### The Target Group

##### Specification of the Target Group Who Will Probably Benefit From KASPAR

Obtaining a clear insight on who would be a suitable target group for KASPAR should be learned from ongoing work and experiences according to the professionals. However, quite some of them immediately had particular children in mind when they were thinking about for whom KASPAR could be of added value. These are some examples that give an indication of children they could see benefiting from KASPAR; i.e. children who:

- have a strong need for proximity and prompting;
- experience difficulties making contact or are anxious in nature to engage in contact with other people;
- have difficulties with the unpredictable nature of people and peers in particular;
- have a kind of urge for ‘safe’ discovery (and like action-reaction interactions);
- might have limited verbal skills or (other) difficulties to express themselves with words;
- have difficulties relating to social-emotional or communicative aspects;
- feel confident and excited using technology;
- seem to be in ‘their own world’;
- are from around 4 years of age and older (depending on their social and cognitive development). Professionals expected that it is not so much the biological age that defines the KASPAR target group, but rather the socio-communicative–cognitive development of the child;
- have a cautious attitude and do not (really) engage in interaction with humans;
- experience problems related to attachment.

##### Specification of Target Group Who Will Probably Not Benefit From KASPAR

Some professionals also described children for who KASPAR would probably not be a success in their eyes. Some children (or adolescents) might not consider KASPAR as being “cool” or may be applicable for young(er) children only. For others KASPAR might not be an appropriate choice because of their high activity level, high ‘aggression’ levels or because they are easily bored.

I showed a picture [of KASPAR] to my son, who has Asperger who said: “why don’t you give me a normal robot that I can use, this is not a real robot but a doll.*—*Adult with autism, who is a professional as well and has a son with autism (Asperger)

#### Environment

Some professionals saw possibilities of using KASPAR in a group setting, for example in the middle of a classroom where all children sit in a circle around the robot. When they suggested individual sessions, they referred to a quiet, calm and peaceful room where there are very little distracting (sensory) stimuli or triggers outside the classroom.

#### Professional

Working with KASPAR demands some requirements from the professional (teacher, therapist) according to the participants:

- professionals need basic instructions on how to operate KASPAR;
- some (not all) professionals need to know how to make new scenario’s using KASPAR’s configuration software or how to fine-tune/modify existing ones.

Professionals with varying backgrounds are proposed to be working with KASPAR as he can be used for different therapy and educational objectives. Professions such as a speech therapist, an occupational therapist, a teacher, pedagogical staff, a physiotherapist, a play therapist, psychologist and also parents have all been suggested as potential end-users. It is important that they have knowledge of and are experienced in working with children with autism and that they can see how to move to transfer and generalisation of required knowledge or skills step by step. Several characteristics and skills have been suggested to be important, such as being very alert and aware, knowledgeable about and attentive to the child and understanding how to dynamically control KASPAR in a proper manner, having an open mind to use new technology, having a positive and enthusiastic attitude and nature, seeing opportunities rather than problems in trying new ways of working with these children, feeling confident that they can work with KASPAR, and last but not least, being able to use their professional intuition and creativity. The professionals expected that the person operating KASPAR is a huge determiner of the success of the interaction and thereby of the intervention. It is recommended that over time children work with different, but not too many professionals (after the child feels at ease) in order to stimulate generalisation and transfer.

It will depend on what you put in as a teacher or therapist. The success of it is not only dependent on the child, it will actually be a result of various skills of the person operating KASPAR.—Teacher at special needs school

I think you ask quite a bit of a teacher working with such a vulnerable target group to let go and try something new. We will have to see what the effects are. Being brave to step back a bit in the interaction and put something else in between.—Teacher at special needs school

#### Intervention Implementation and Integration into Current Practices

##### Integration in the Individualised Education/Therapy Plan

Integration was mentioned very often during the sessions as being crucial for a proper implementation of robot-mediated interventions, meaning that KASPAR should not function as a standalone platform, but its use should be integrated in common practices. It is crucial to integrate the work with KASPAR in the overall education/therapy plan of the children. Often schools or care organisations work with an individualised therapy/education/care plan that describes what learning objectives the focus is on for a particular child for the upcoming weeks or months. Based on this personalised plan, education or therapy actions will be taken by professionals. Each child has his/her own plan which is updated regularly. Professionals indicated that when KASPAR is used therapeutically, it has to be part of a conscious decision of knowing for which children KASPAR will be used, what objective(s) to work on, how, when, where, how long and often, and by whom. This all has to be documented (and introduced, executed, evaluated) in the individualised plan as any other intervention. KASPAR is considered to be simply another means, a tool that professionals have at their disposal that is imbedded in the plan and protocols, not used in an ad hoc manner. It is envisioned that there will be kind of “KASPAR scenario library” (containing varying scenario’s, behaviours, sounds) from which can be chosen depending on the needs of the particular child at that moment.

Finally, time is crucial. Professionals stressed the need to give the children the time they need to get used to KASPAR. Changes are difficult for children with autism and they normally take quite some time to accustom to new situations. Time is also required for the professionals who have to learn how to work with KASPAR.

##### Integration in Organisation and Professional Levels and Connecting to Parents

Professionals argued that on an organisational or management level a vision needs to be developed and deployed on how to implement and use KASPAR. This plan and strategy prevents KASPAR to be used in an ad hoc manner without relevant effects. This entails aspects such as ensuring proper (internal and external) communication, training, planning, and adequate allocation of resources.

It will be crucial to have realistic expectations of this [KASPAR interventions], that we see it as a tool and not more than that. This is important because we do not want to present it as thé Holy miracle solution that makes promises but then creates a disappointment.—ASD therapy expert and coach

Professionals mentioned it is important to inform parents of the children that will be interacting with KASPAR and maybe even (actively) involve them and ask their feedback or help in optimising the use of KASPAR for their children. Professionals suggested that parents might provide situations that are difficult for their children that can function as training situations in a KASPAR session. Moreover, it was suggested that possibly on a longer term (some) parents might also become users of KASPAR in the home environment. Furthermore, according to professionals it was crucial that there is a kind of feedback and learning loop between and from the professionals who work with KASPAR to the rest of the team to share the experiences and ideas.

#### Phase in the Intervention Trajectory

Participants distinguished between aspects that are important factors in using KASPAR in different phases in the intervention trajectory: training, introduction, session preparation, actual usage and evaluation. They indicated that the person(s) who will work with KASPAR and the children receive a proper training on how to configure, prepare, and use the robot. According to them, part of this training should include a number of hours practice with KASPAR before they are actually going to use it in a session with children. Training should contain a technical component of how to set-up and operate the robot. Besides this, participants suggested that also a social interaction component is crucial for developing a feeling and skill to ‘read’ the child and being able to provide prompt KASPAR (re)actions. In their view, training and practicing are crucial to be able to create success experiences, both for the children and the professionals.

You can place KASPAR in a room and do nothing, the child will notice him, and you’ll see immediately if there is interaction or not…because if he will be introduced formally then you bring in the anxiety factor as well for something unknown, while if you can explore yourself you know best what you want and don’t want—Adult with ASD

For the *introduction* many professionals suggested to place KASPAR on a table in a room and let the children approach him in their own pace and own preferred way. They compared introducing KASPAR with mastering the art of simplicity in the beginning; exposing a child to a fully equipped and completely extensively programmed robot will probably create adverse effects. Rather, they suggested to dose more interaction/initiative from KASPAR in a slow and step by step way, in a manner that matches the needs of the child. They stressed the importance that the child has the freedom and time to explore KASPAR for him/herself and decides if and how there will be interaction in the first moments. The amount and intensity of the triggers (e.g. sounds, movements, actions, utterances) that KASPAR gives shall be gradually adjusted by the professional according to the needs and capacities of the child. Participants reported that, for some children this might be a matter of some days, while others may need weeks or even months to get familiar and at ease with KASPAR. Others might not like interaction with KASPAR at all, which is fine as well.

After the introduction and training took place, participants stressed a number of important aspects in preparing for each session. When getting ready for the day the *session preparation* will be done in which the professional decides on what objectives (s)he will work on that day with KASPAR for whom and what scenarios are needed. Fine-tuning and adjusting scenario’s will be done during the preparation phase as well, according to the needs of the children that will take part in the KASPAR sessions. Professionals suggested to create and use a dedicated symbol for KASPAR in line with the Picture Exchange Communication System (PECS) method that these children work with in their day structure. The KASPAR symbol would then be placed in the day structure/-programme of the child, so that the child also knows that there will be a session with KASPAR that day and when. Besides this, they stressed that in the planning of the professionals it is assured that also other professionals are available to work with the group of children while a trained KASPAR professional works with one or a small group of children in a dedicated KASPAR session.

During the actual usage, conscious experimentation and adaptation will be needed in the views of the participants to learn what works for a particular child and what not once the child received a basic KASPAR training. They expected that the success of the intervention will depend on how the child reacts, but also very heavily on the way the professional is able to control KASPAR in the interaction. Professionals should guide the children through the interaction, build up the sessions in a pace that matches the needs and capabilities of the child, and be attentive to prevent sudden abrupt moves, actions or sounds of KASPAR. Participants expected that the duration of a session will vary per child and possibly also per phase in the intervention. To increase chances for transfer/generalisation, KASPAR should be used in different rooms, according to participants, at different moments and by different people at appropriate times.

Creating (regular) evaluation moments is suggested, both with the children to learn more about their experiences as well as with professionals using KASPAR and their management. Possibly one can consider recording some sessions in order to learn from experiences. Moreover, celebration of success moments was expected to be crucial as well.

#### Session Characteristics

Professionals saw possibilities for using KASPAR in different kinds of sessions; individual sessions like a 1-1-1 setting (child-KASPAR-professional), a group session (in a group interaction in small classroom for example), or in a 2/3-1-1 (2/3 children—KASPAR—professional) setting. With very young children KASPAR might be used in a ritual in a group to start the day; this creates a safe and predictable moment. For a large number of children they expected to be working in individual sessions (since these children have difficulties functioning in group settings).

I feel very curious, you will have children with whom I want to work very goal oriented, and there are also kids of which I simply would love to see their reaction.—Teacher at special needs school

Another distinction that was made is the degree of structure in the session; it can be a rather free explorative session, semi-structured, or structured. In all three cases, professionals stressed that if the child is not interested (anymore) in KASPAR, the session will be stopped, persuasion of the child to continue is absolutely out of the question.

With respect to the duration of KASPAR sessions, professionals suggested to make it rather short time frames to match the attention span of the children with autism (i.e. 10–15 minutes, but maximum 30 minutes).

### Intervention Template

**Fig. 2:**
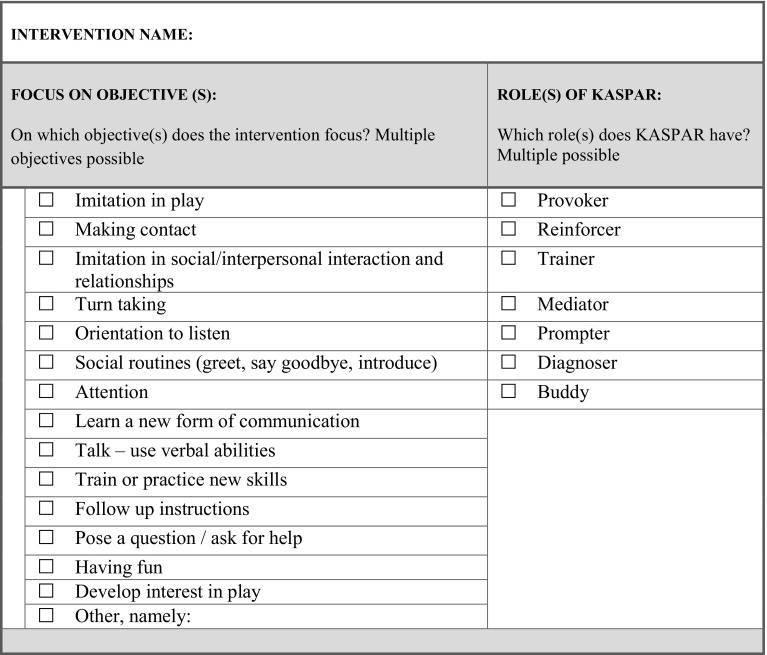
Template to describe robot mediated intervention—objectives and robot roles

Insights gained from the focus groups as well as findings (educational/therapy objectives) from previous work (Huijnen et al. 2016b) allowed to create the robot mediated intervention template. Important elements that were suggested by the professionals to be included in a robot mediated intervention template are described. Firstly, they indicated that the intervention should have a name and therapy and/or educational objectives will be addressed by this intervention should be selected. Furthermore, one or more roles for KASPAR are chosen (see Fig. 2).

**Fig. 3:**
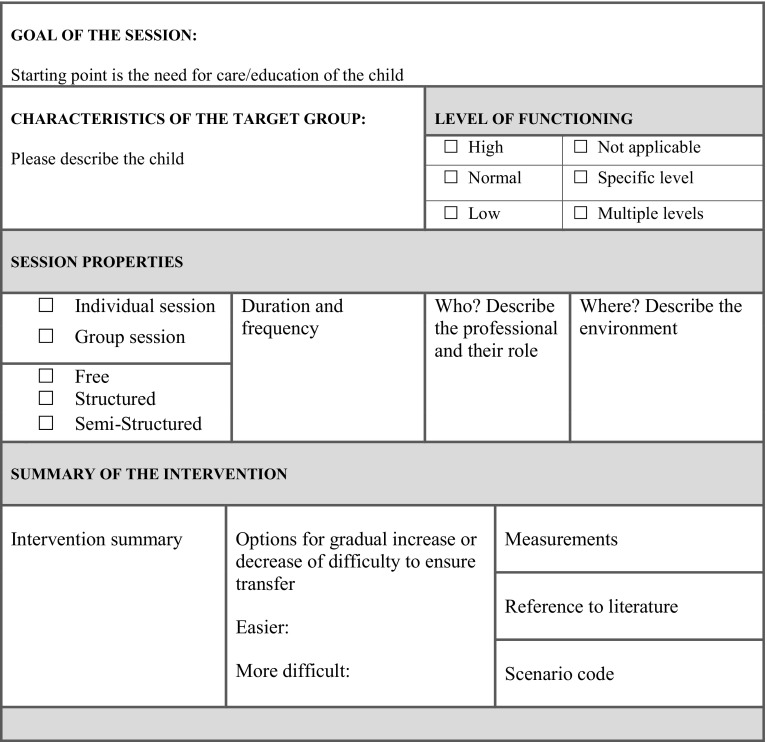
Template to describe robot mediated intervention—intervention description

**Fig. 4:**
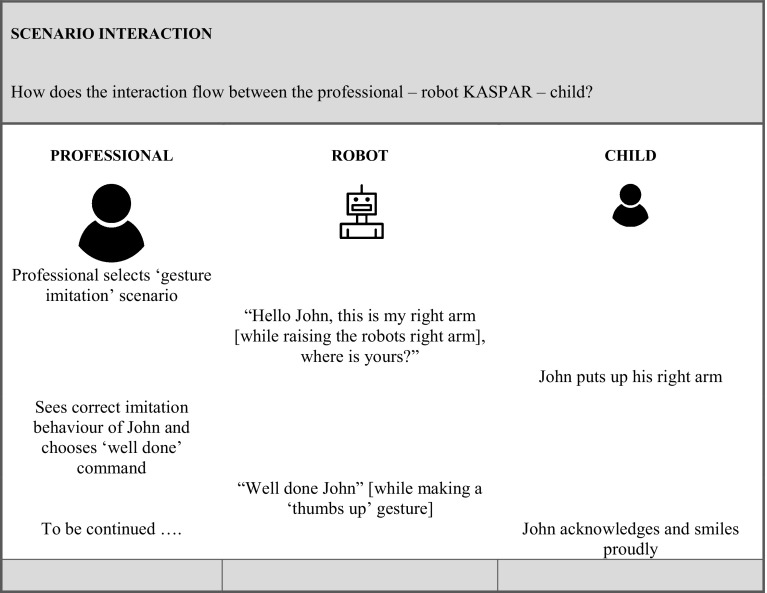
Template to describe robot mediated intervention—intervention interaction flow

Subsequently, participants stressed that the goal of the intervention should be clear and adequately described, which is driven by the specific needs of the particular child. Furthermore, they highlighted the importance of creating a detailed characterisation of the child as well as of his/her level of functioning which is needed to get a better idea of the target group for this particular intervention (see Fig. 3). The session characteristics that were mentioned by the participants in “Requirements for KASPAR Mediated Interventions” are then described, a short summary is given, and ways to work towards transfer are outlined and how to measure effects (in ‘measurements’). Finally, Fig. 4 shows how the actual interaction flow between the professional, KASPAR, and the child will go in a stepwise approach for a particular scenario.

This template is a result created based on the previous focus group findings and used as input for the co-creation sessions in which a number of new KASPAR interventions were made.

### Co-created Interventions

A total number of 10 new KASPAR mediated interventions were created during the co-creation sessions. Table 4 lists all the generated ideas shortly. This paper presents one example in detail (see “Appendix 2”). The ASD objectives overview show the ideas; this is a categorisation of domains of therapy and educational objectives that are important for children with ASD as presented in (Huijnen et al. 2016b) “Appendix 1”also presents this overview. The Child and Youth version of the International Classification of Functioning, Disability and Health of the WHO, more commonly known as ICF-CY, functioned as a classification framework (World Health Organization 2007).

**Table 4:** Generated KASPAR interventions during co-creation sessions

Intervention idea	Domain
KASPAR helps to learn making eye contact	Communication
KASPAR helps to learn how to greet in the morning 1	Social interaction and interpersonal relations
KASPAR helps to learn how to greet in the morning 2	
KASPAR helps to learn to greet when entering a room	
KASPAR helps to improve/stimulate a play attitude	Play
KASPAR helps with making homework	Preschool skills
KASPAR supports with executing a task	
KASPAR helps in self-reflection	Emotional wellbeing
KASPAR helps to create peace of mind	
KASPAR helps to be able to have breakfast independently	Self-care, independent living
	Sensory experiences and coping
	Functioning in daily reality
	Motor experiences and skills

One example of these intervention ideas is “KASPAR supports with executing a task”. It addresses the therapeutic and educational objectives of “orientation to listen”, “follow up instructions” and “pose a question/ask for help”. KASPAR takes the role of a “provoker”, “reinforcer”, “trainer”, and a “prompter”. The intervention is applied individually in a structured manner. Often, children with ASD experience difficulties with taking initiative and performing tasks independently. In classes, often picto’s (visual symbols part of the PECS method) are used that show an activity/task and these actual activities/tasks are stored in separate baskets. In this intervention KASPAR will help the children to work more independently using this picto/basket system by giving step by step instructions, prompts for working on the task and positive reinforcements to reward their behaviour. The entire intervention (including all the description details and interaction flow presented in the template) can be seen in “Appendix 2”.

**Fig. 5:**
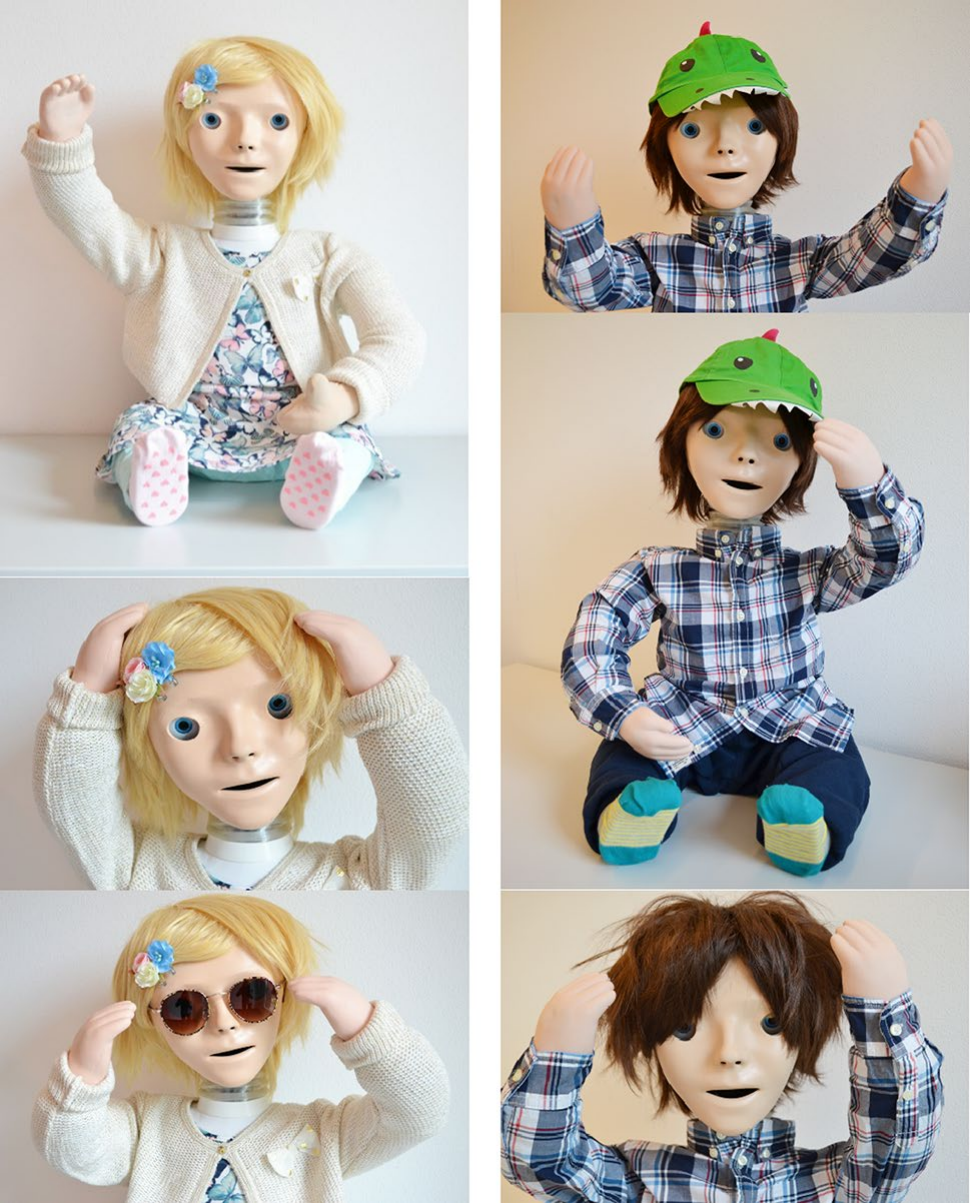
KASSY (girl version, *left*) and KASPAR (boy version, *right*)

Based on the input gathered at the session, the intervention template was further refined (e.g. use of better wording) and also a girl version of robot KASPAR was created since multiple professionals indicated this would be desirable (see Fig. 5).

**Fig. 6:**
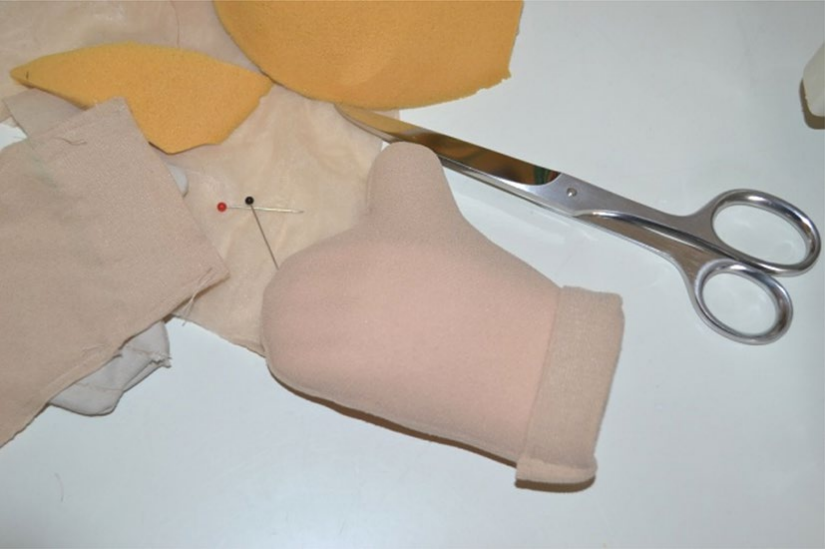
Creating KASPAR’s thumb

**Fig. 7:**
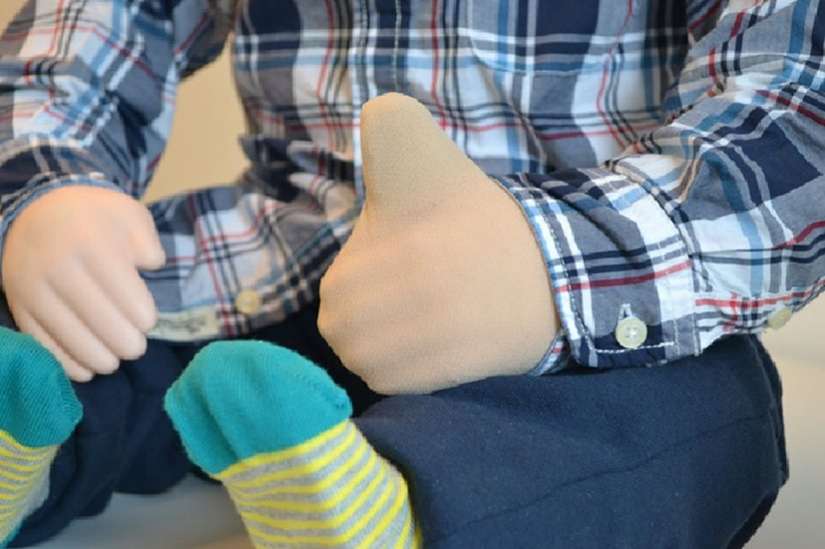
Thumb up on hand

Moreover, participants suggested that KASPAR should also be able to give a “thumbs up” to reward children in a non-verbal manner. This was created as well (see Figs. 6, 7).

## Discussion and Conclusion

The aim of this study was to gain understanding on how to implement robots in interventions for children with ASD. Highlighting a case for the use of robots in interventions for this target group was made decades ago, but actual use is still scarce. We intended to contribute to an increase of awareness and insight on how to actually embed robots in current education and/or therapy practices. The approach was to involve a large number of ASD practitioners and other stakeholders in the field, including people with ASD and parents of children with ASD, in focus groups as well as in co-creation sessions.

Results indicate that besides requirements related to the robot itself such as appearance, the use of voice and sound, the operation, the robot’s behaviour and a robots attributes/toolbox, many other factors need to be taken into account. Personalisation to the needs of the individual child at hand is the main message. Keeping that in mind, it is clear that the look and behaviour of the robot is a crucial aspect, but also the role of the professional, the environment, and educational and organisational integration will be key in actual implementation in practice.

These results are in line, yet go beyond other published work on robots for children with autism that often tends to focus on human robot interaction matters. The utmost importance as well as a sense of urgency for robot research to be sensitive and adhere to end-users’ requirements and to grow closer towards clinical integration into robot-mediated-interventions has been clearly argued in a number of recent publications (Cabibihan et al. 2013; Diehl et al. 2012; Wainer et al. 2014a). To date, only few studies are published on systematically eliciting and describing requirements for robot-assisted interventions and how to actually embed robots in current practices. By intensively involving and co-creating interventions together with professionals and other stakeholders we aimed to increase chances for clinical relevance and uptake and overcome typical barriers for robot mediated interventions to reach clinical applicability as stated by Begum et al. (2016). The contents of the developed intervention template in this study cover (and to some extend expands to) Begum et al’s elements to be included in an EBP.

The adoption of this iterative multidisciplinary co-creation approach is expected to contribute to qualitative and meaningful robot interventions. This study provides a tool, the robot intervention template, for systematically developing and implementing new robot interventions and can contribute to an increase in awareness and the creation and uptake of robot assisted interventions for children with autism. It can be used by both professionals, stakeholders and engineers, for many different robot platforms, not only for KASPAR.

Although the study has reached its goals, some limitations should be taken into account. First of all only participants from The Netherlands were included in the study which might hinder generalisability because other countries might have different practices in place regarding care or education for children with ASD. On the other hand, it is expected that when considering the heterogeneous nature of ASD, involving more than 75 participants covers a wide range of knowledge and experiences on ASD. Additionally, ideally, we would have immediately implemented the co-created interventions in KASPAR and would have like to test it in practice rather quickly to evaluate the applicability of these interventions. However, technical implementation of the interventions in the robot, practically training professionals to operate the robot themselves as well as assuring approval of medical/ethical committees is needed before we can actually test these interventions with children with autism.

The heterogeneous nature of ASD causes a demand for different and multiple treatment or interventions for different children. There seems to be consensus that a “one-size-fits-all” solution for all children with ASD does not exist (Stahmer et al. 2011). There is no such thing as “the average disabled person” or “average context” (De Couvreur and Goossens 2011). This makes customisable robot-assisted interventions a strong appropriate candidate due to their adaptability and capacities for tailored personalisation. Whether or not a robot intervention will be a success in practice will depend on the dynamic interplay of many (changing) factors, not just on the availability of a stable technical robot platform. This study aimed to shed more light in the relevant aspects of this interplay. Future research entails conducting a pilot and an effect study in which professionals actually use these KASPAR interventions in practice with children with ASD, so that actual effects on both the professionals and children can be assessed.

## Figures and Tables

**Fig. 8:**
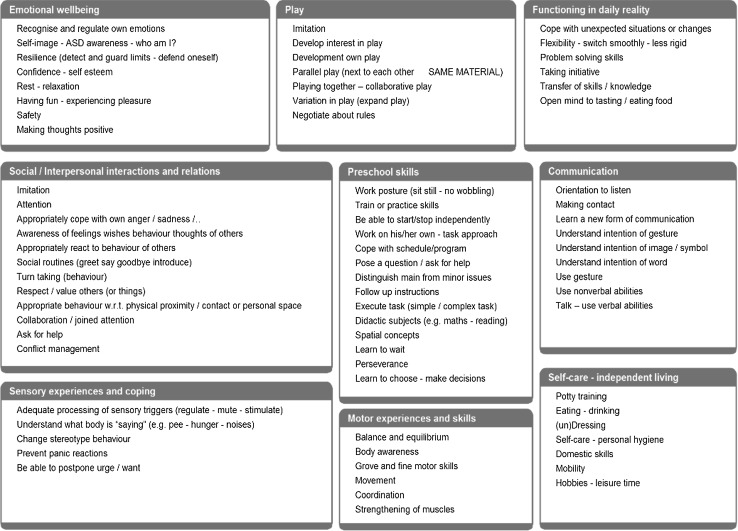
Complete overview of therapy and education objectives for children with ASD (Huijnen et al. 2016b)

**Fig. 9:**
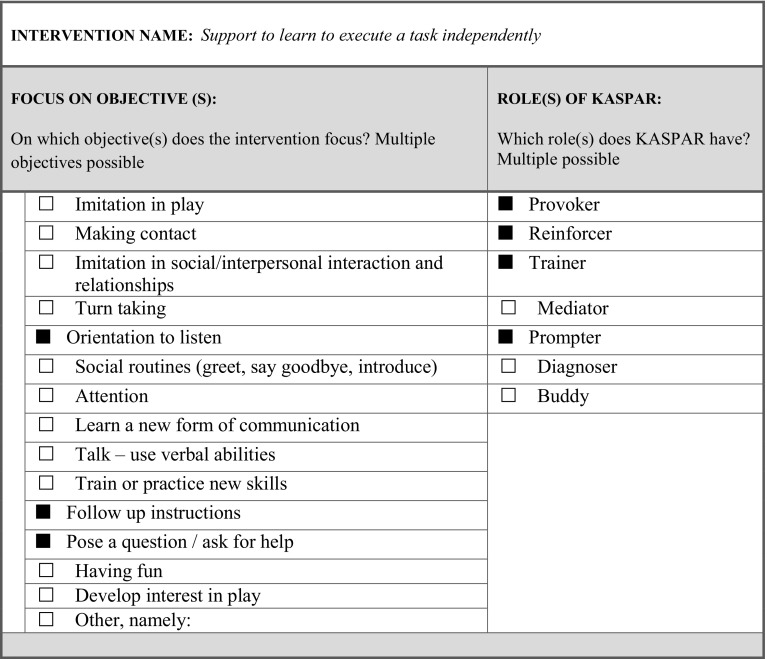
Objectives and roles of example intervention

**Fig. 10:**
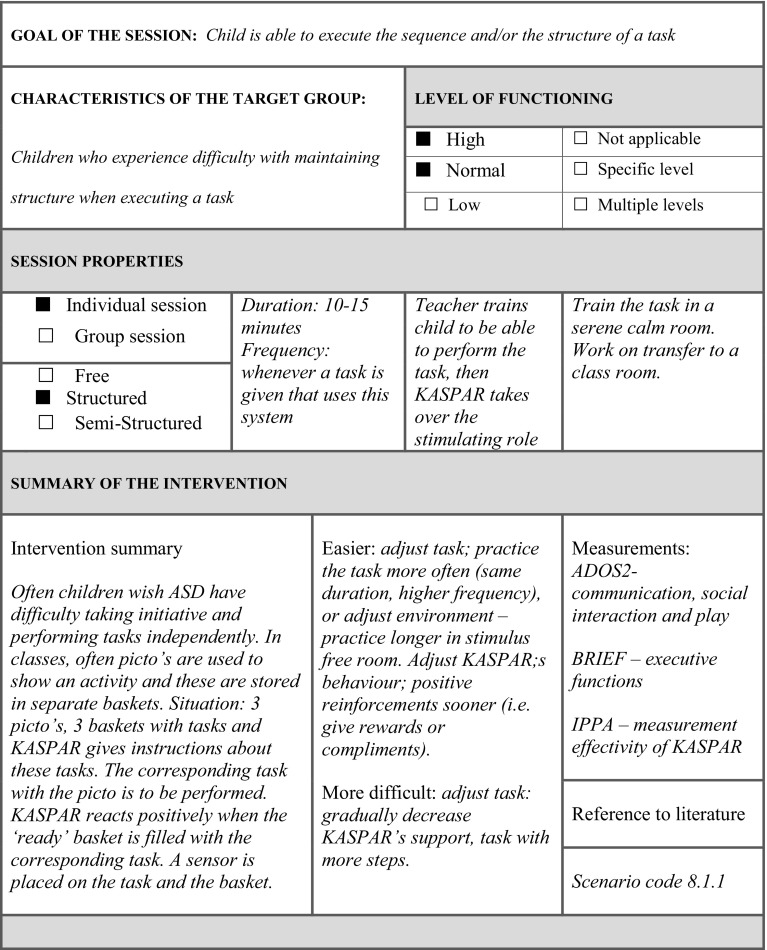
Details of intervention description

**Fig. 11:**
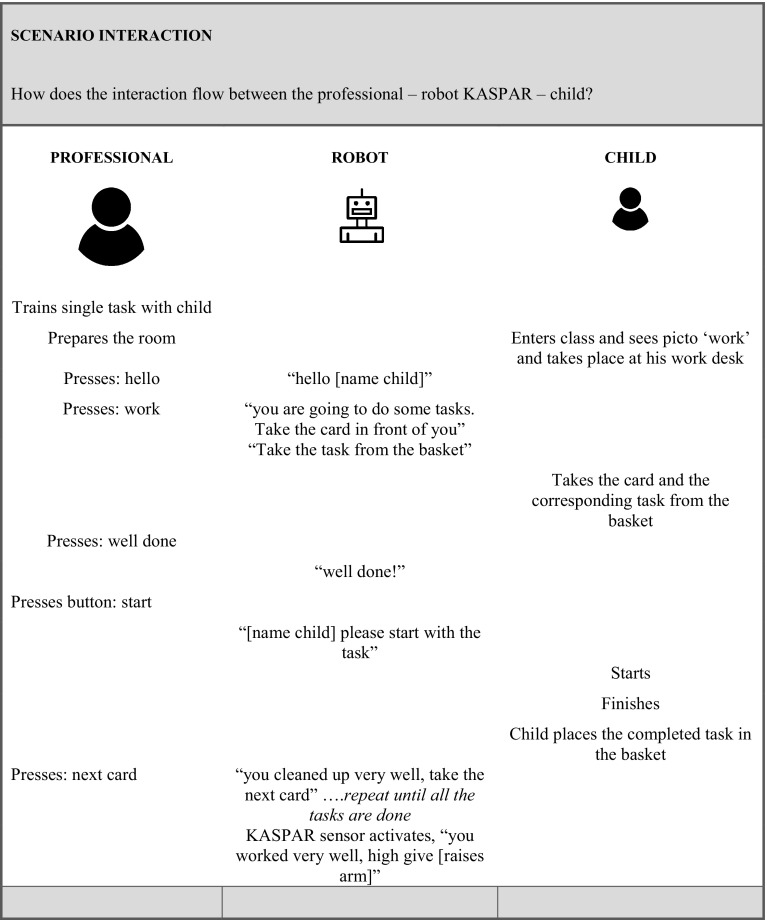
Interaction flow between professional—KASPAR—child

## Appendix

### Appendix 1: ASD Therapy and Educational Objectives Overview

See Fig. 8.

### Appendix 2: Detailed Intervention Idea in Template

See Figs. 9, 10, 11.
